# Low FVC/TLC in Preserved Ratio Impaired Spirometry (PRISm) is associated with features of and progression to obstructive lung disease

**DOI:** 10.1038/s41598-020-61932-0

**Published:** 2020-03-20

**Authors:** Spyridon Fortis, Alejandro Comellas, Victor Kim, Richard Casaburi, John E. Hokanson, James D. Crapo, Edwin K. Silverman, Emily S. Wan

**Affiliations:** 1 Center for Access & Delivery Research & Evaluation (CADRE), Iowa City VA Health Care System, Iowa City, IA USA; 2 Division of Pulmonary, Critical Care and Occupational Medicine, University of Iowa Hospital and Clinics, Iowa City, IA USA; 3 Department of Thoracic Medicine and Surgery, Lewis Katz School of Medicine at Temple University, Philadelphia, PA USA; 4 Lundquist Institute for Biomedical Innovation at Harbor-UCLA Medical Center, Torrance, CA USA; 5 Department of Epidemiology, Colorado School of Public Health, University of Colorado Denver, Denver, CO USA; 6 Department of Medicine, National Jewish Health, Denver, CO USA; 7 Channing Division of Network Medicine, Brigham and Women’s Hospital, Boston, MA USA; 8 VA Boston Healthcare System, Jamaica Plain, MA USA

**Keywords:** Medical research, Biomarkers, Epidemiology, Outcomes research

## Abstract

One quarter of individuals with Preserved Ratio Impaired Spirometry (PRISm) will develop airflow obstruction, but there are no established methods to identify these individuals. We examined the utility of FVC/TLC in identifying features of obstructive lung disease. The ratio of post-bronchodilator FVC and TLC_CT_ from chest CT (FVC/TLC_CT_) among current and former smokers with PRISm (FEV_1_/FVC ≥ 0.7 and FEV1 < 80%) in COPDGene was used to stratify subjects into quartiles: very high, high, low, and very low. We examined the associations between FVC/TLC_CT_ quartiles and (1) baseline characteristics, (2) respiratory exacerbations, (3) progression to COPD at 5 years, and (4) all-cause mortality. Among participants with PRISm at baseline (n = 1,131), the very low FVC/TLC_CT_ quartile was associated with increased gas trapping and emphysema, and higher rates of progression to COPD at 5 years (36% versus 17%; p < 0.001) relative to the very high quartile. The very low FVC/TLC_CT_ quartile was associated with increased total (IRR = 1.65; 95% CI [1.07–2.54]) and severe (IRR = 2.24; 95% CI [1.29–3.89]) respiratory exacerbations. Mortality was lower in the very high FVC/TLC_CT_ quartile relative to the other quartiles combined. Reduced FVC/TLC_CT_ ratio in PRISm is associated with increased symptoms, radiographic emphysema and gas trapping, exacerbations, and progression to COPD.

## Introduction

Among individuals in the general population with at least 10 pack-years of cigarette smoking who undergo spirometry, 12% have a post-bronchodilator FEV_1_% predicted below 80% with a FEV_1_/FVC ratio ≥ 0.7^1^. This “non-specific” spirometric pattern, commonly referred to as “restrictive”, has been designated as Preserved Ratio Impaired Spirometry (PRISm)^2–7^. Smokers with PRISm have higher cumulative smoking exposure, reduced exercise capacity, and increased mortality compared to smokers with normal lung function^1,2,7^. PRISm is also associated with thicker airway walls relative to smokers with normal spirometry^2^. PRISm individuals share features with COPD patients, and a quarter of subjects with PRISm eventually develop COPD^1,2^. The range of post-bronchodilator FEV_1_% predicted and percent emphysema on CT among PRISm subjects is wide, indicative of a heterogeneous population^2^. Clustering analysis identified a “PRISm-COPD” subgroup with lower FEV_1_/FVC ratio and higher radiologic CT emphysema relative to the rest of PRISm subjects^3^. This subgroup may represent individuals with early or occult obstructive lung disease who do not meet current diagnostic thresholds for airflow limitation.

In PRISm, total lung capacity (TLC) may help distinguish a restrictive from an obstructive ventilatory defect, according to the American Thoracic Society-European Respiratory Society (ATS- ERS) 2005 guidelines^8^. However, a true “restrictive disease” is very unlikely in individuals with risk factors for obstruction lung disease, no interstitial lung disease and unremarkable body mass index (BMI). In COPDGene, the prevalence of PRISm is 12% despite the fact that participants with interstitial lung disease were excluded and body mass index (BMI) in PRISm individuals was slightly higher than the BMI in smokers with normal lung function^1,2^. In addition, a single center study showed that among individuals with PRISm and TLC above the lower limit of normal (LLN), only 26% had a clinical diagnosis of obstructive lung disease^9^. Moreover, only 15% of those with PRISm and TLC > LLN develop obstructive spirometry over a median follow-up time of 3 years^10^. Currently, there is no available diagnostic test in clinical practice to identify which patients with PRISm may have features classically associated with obstructive lung disease.

In obstructive lung diseases, residual volume (RV) may increase at the expense of FVC with total lung capacity (TLC) remaining normal^11^. Conversely, RV may increase with a preserved FVC resulting in an increased TLC. Both processes result in reduced FVC/TLC ratio which may antedate the development of obstruction diagnosed using standard FEV_1_/FVC criteria. A disproportionate decrease of FVC relative to TLC may occur in patients with a restrictive ventilatory defect that coexists with obstructive lung disease^12^. Thus, FVC/TLC represents a composite measure that may be able to identify an occult obstructive ventilatory defect. FVC can be readily obtained from spirometry, while TLC, typically assessed by plethysmography, can also be quantified using an inspiratory chest CT (TLC_CT_), with prior studies demonstrating strong correlations with the plethysmography results^13,14^.

We hypothesized that reduced FVC/TLC ratio in PRISm is associated with clinical, functional and radiographic features of obstructive lung disease, acute respiratory events and increased mortality, and progression to COPD. To investigate our hypothesis, we examined current and former smokers with PRISm enrolled in the COPDGene study.

## Methods

### Data collection

We analyzed data from participants in the COPDGene study, an ongoing study conducted at multiple clinical centers through the United States (http://www.copdgene.org/). Participants were current and former smokers with ≥10 pack-years of smoking who self-identified as non-Hispanic whites (NHW) or African Americans (AA) and were between the ages of 45–80 years at enrollment. The institutional review boards at each participating center approved the study protocol and written informed consent was obtained from all participants. Details of the study protocol have been published previously^15^. Briefly, participants completed a modified American Thoracic Society Respiratory Epidemiology questionnaire, St. George’s Respiratory Questionnaire (SGRQ), and 6-minute walk test (6-MWT) at the enrollment visit. Dyspnea was assessed using the modified Medical Research Council (mMRC) scale. Participants performed pre- and post-bronchodilator spirometry. The complete study protocols were performed in accordance with the relevant guidelines and regulations of American Thoracic Society–European Respiratory Society (ATS-ERS)^16^. Volumetric chest CT scans were obtained at TLC_CT_ (maximal inspiration) and at functional residual capacity (FRC_CT_) (end-tidal expiration) using multidetector CT scanners^15^. FRC and TLC% predicted were calculated based on the predicted values^17^. Percent emphysema and gas trapping were quantified using 3D Slicer software (www.airwayinspector.org)^15^.

We included participants with PRISm at enrollment. We excluded individuals with significant interstitial lung disease or bronchiectasis on chest CT, those with missing post-bronchodilator spirometry or TLC_CT_ measurements at baseline, and participants with post-bronchodilator FVC > TLC_CT_ at enrollment. Approximately 5 years after the enrollment visit, participants were invited for a follow-up visit that included a repeat spirometry and chest CT. Respiratory exacerbation data were collected prospectively after enrollment. Participants were contacted every 6 months after enrollment and completed a standardized questionnaire regarding respiratory exacerbations through the Longitudinal Follow Up program. Vital status was also ascertained using information from the social security death index and the Longitudinal Follow Up program.

### Definitions and outcomes

PRISm was defined as post-bronchodilator FEV_1_ < 80% predicted and FEV_1_/FVC ≥ 0.7. COPD was defined as post-bronchodilator FEV_1_/FVC < 0.7. The FVC/TLC_CT_ ratio at enrollment was calculated using post-bronchodilator FVC (in liters) from spirometry, while TLC_CT_ was measured from volumetric inspiratory chest CT scans.

Co-morbidities and medication usage were self-reported. Percent emphysema was defined by using the percentage of lung volume at TLC_CT_ with attenuation less than −950 Hounsfield units (HU)^15^. Gas trapping was quantified as the percentage of lung volume at FRC with attenuation values less than −856 HU^15^. Parametric response mapping analysis was performed on paired registered inspiratory and expiratory images to distinguish functional small airways disease (PRM^fSAD^) from emphysema by Imbio LLC (Minneapolis, MN) using lung density analysis software^18^. As previous described^19^, we defined PRM^fSAD^ as the percentage of lung with evidence of gas trapping *not* due to emphysema (i.e. areas of lung with attenuation < −856 HU on expiration minus area of lung with attenuation < −950 HU on inspiration).

Change in FEV_1_ between enrollment and 5-year follow up visit was calculated using post-bronchodilator spirometry. Exacerbations were defined as episodes of worsening respiratory symptoms requiring use of antibiotics and/or systemic steroids. Severe exacerbations were defined as those requiring hospitalizations or emergency room visits. Other variable definitions have been previously described^15^.

### Statistical analysis

We stratified PRISm participants at the enrollment visit into quartiles by FVC/TLC_CT_: very high, high, low, and very low. We compared the characteristics of PRISm individuals at the enrollment visit, rates of progression to COPD at the 5-year follow-up visit, and exacerbations over the time between the FVC/TLC_CT_ quartiles. We used Spearman’s rank correlation to examine changes in continuous variables with increasing FVC/TLC_CT_. We used the Cochran Armitage trend test to examine proportion changes with increasing FVC/TLC_CT_ quartile.

We created multivariable logistic and linear regression models with chronic bronchitis, mMRC and SGRQ scores, radiographic measures and 6-MWT distance at the enrollment visit as the dependent variable (outcome) and post-bronchodilator FVC/TLC_CT_ quartile at the enrollment as the independent variable (predictor). All models included the following covariates: age and current smoking status at enrollment, gender, race, pack-years smoked, body mass index (BMI), history of asthma and congestive heart failure. There were no missing values in any of the covariates. We also performed a multivariable linear and logistic regression analysis with change in FEV_1_, 6-MWT distance, radiographic measurements, and progression to COPD at the follow-up visit as the dependent variable (outcome) and post-bronchodilator FVC/TLC_CT_ quartile at enrollment as the independent variable (predictor). We included the following covariates in these models: age and current smoking status at enrollment, gender, race, pack-years smoked, body mass index (BMI), history of asthma and congestive heart failure, and change of BMI between enrollment and follow-up visit.

For the exacerbation analysis, we created zero-inflated negative binomial models which included adjustment for age and current smoking status at enrollment, gender, race, pack-years smoked, BMI, chronic bronchitis, history of asthma and congestive heart failure. There were no missing values in any of the covariates. Follow-up time was included as an offset in the models as previously described^20^.

We used Cox proportional hazard regression models to examine the association between post-bronchodilator FVC/TLC_CT_ quartile with all-cause mortality. Models included the following covariates: age, gender, race, smoking status, smoking pack-years, BMI, diabetes, history of asthma and congestive heart failure. There were no missing values in any of the covariates.

In sensitivity analyses, we repeated selected analyses using PRISm defined as post-bronchodilator FEV1 < LLN with a post-bronchodilator FEV1/FVC ≥ LLN and COPD defined as post-bronchodilator FEV1/FVC < LLN using the NHANES III reference values^21^. All statistical analyses were conducted using R statistical software (http://www.r-project.org/) using the following R software packages: ‘dunn.test’, ‘FSA’, ‘pscl’, ‘MASS’, ‘AER’, ‘survival’, and ‘DescTools’.

### Ethics approval

The institutional review boards at each participating center approved the study protocol. Details of the study protocol have been published previously^16^.

## Results

Of 10,199 COPDGene participants with at least 10 or more pack-years of smoking and no significant interstitial lung disease or bronchiectasis, 1,260 of them had PRISm at the enrollment visit. After excluding one individual with no available post-bronchodilator spirometry, 121 with no TLC_CT_ measures and 7 individuals with FVC > TLC_CT_, 1,131 participants were included in the analysis. The median value of FVC/TLC_CT_ was 0.59 (IQR = 0.53–0.66). Of these 1,131 participants, 617 of them had acceptable spirometry measurements at the 5-year follow-up visit, 967 had available data regarding respiratory exacerbations, and 960 had vital status data available.

### Baseline characteristics at the enrollment visit (n = 1,131)

Table 1 shows the characteristics of participants by FVC/TLC_CT_ quartile. Age, BMI, pack-years smoking exposure, mMRC and SGRC scores, % emphysema and gas trapping, and % functional small airways disease increase with decreasing FVC/TLC_CT_. An increased proportion of females and decreased proportion of African Americans were associated with decreasing FVC/TLC_CT_. Participants in the lower FVC/TLC_CT_ quartiles a higher prevalence of comorbidities.

**Table 1:** Baseline characteristics of smokers with preserved ratio impaired spirometry across post-bronchodilator forced vital capacity/total lung capacity ratio (FVC/TLC_CT_) quartiles (n = 1,131).

FVC/TLC Quartile	High air trapping → Low air trapping				P for trend
	Very Low quartile (n = 283)	Low quartile (n = 283)	High quartile (n = 282)	Very High quartile (n = 283)	
**FVC/TLC**_**CT**_	<**0.53**	**0.53–0.59**	**0.59–0.66**	**>0.66**	
**Age, y** ± **SD**	62.83 ± 8.82	57.68 ± 7.34	55.70 ± 6.96)	52.84 ± 6.27	<0.001
**Female, n (%)**	186 (65.7%)	169 (59.7%)	143 (50.7%)	115 (40.6%)	<0.001
**African American, n (%)**	86 (30.4%)	89 (31.4%)	126 (44.7%)	174 (61.5%)	<0.001
**BMI, Kg/m**^**2**^ ± **SD**	32.99 ± 7.42	32.84 ± 7.50	30.24 ± 6.88	31.05 ± 6.97	<0.001
**Pack-Years** ± **SD**	49.46 ± 28.69	43.51 ± 22.51	39.12 ± 20.08	38.06 ± 22.63	<0.001
**Active Smoker, n (%)**	154 (54.4%)	162 (57.2%)	180 (63.8%)	213 (75.3%)	<0.001
**Chronic Bronchitis, n (%)**	53 (18.7%)	54 (19.1%)	52 (18.4%)	42 (14.8%)	0.23
**mMRC** ± **SD**	1.70 ± 1.47	1.56 ± 1.44	1.21 ± 1.37	1.44 ± 1.50	<0.001
**SGRQ** ± **SD**	32.91 ± 22.69	30.06 ± 23.29	24.50 ± 20.54	29.71 ± 23.78	<0.001
**Asthma, n (%)**	75 (26.5%)	64 (22.6%)	51 (18.1%)	60 (21.2%)	0.064
**CHF, n (%)**	21 (7.4%)	17 (6.0%)	5 (1.8%)	8 (2.8%)	0.001
**DM, n (%)**	75 (26.5%)	75 (26.5%)	44 (15.6%)	42 (14.8%)	<0.001
**HTN, n (%)**	150 (53.0%)	151 (53.4%)	133 (47.2%)	120 (42.4%)	0.004
**CAD, n (%)**	25 (8.8%)	32 (11.3%)	12 (4.3%)	10 (3.5%)	<0.001
**OSA, n (%)**	68 (24.0%)	61 (21.6%)	56 (19.9%)	38 (13.4%)	0.002
**CVA, n (%)**	15 (5.3%)	10 (3.5%)	7 (2.5%)	7 (2.5%)	0.049
**LAMA, n (%)**	33 (12.1%)	18 (6.5**%**)	18 (6.5**%**)	15 (5.4**%**)	0.005
**ICS, n (%)**	19 (7.0**%**)	19 (6.8**%**)	12 (4.4**%**)	13 (4.6**%**)	0.131
**LABA, n (%)**	7 (2.6**%**)	1 (0.4**%**)	1 (0.4**%**)	4 (1.4**%**)	0.2482
**ICS/LABA, n (%)**	59 (21.6**%**)	36 (12.9**%**)	22 (7.9**%**)	24 (8.6**%**)	<0.001
**Post-FEV1%** ± **SD**	65.74 ± 9.65	71.33 ± 7.32	72.04 ± 6.54	73.02 ± 5.92	<0.001
**Post-FVC%** ± **SD**	66.70 ± 10.07	72.47 ± 7.68	73.47 ± 7.58	75.05 ± 7.25	<0.001
**BDR, n (%)**	40 (14.4%)	41 (14.6%)	30 (10.8%)	47 (16.8%)	0.71
^§^**% Emphysema** ± **SD**	2.02 ± 3.32	1.66 ± 2.92	1.48 ± 2.00	1.07 ± 1.53	<0.001
^§^**% Gas trapping** ± **SD**	12.48 ± 8.64	9.06 ± 7.48	8.19 ± 6.65	7.50 ± 5.82	<0.001
^‡^**PRM**^**fSAD**^**, % ± SD**	14.63 ± 9.87	10.60 ± 8.65	10.37 ± 9.24	10.13 ± 8.65	<0.001
^§^**FRC**_**CT**_**%** ± **SD**	97.25 ± 18.17	87.62 ± 14.63	80.89 ± 13.00	75.25 ± 12.36	<0.001
**TLC**_**CT**_ **%** ± **SD**	90.20 ± 13.58	85.82 ± 9.80	77.77 ± 9.48	68.96 ± 9.02	<0.001
^**#**^**6-MWT, meters** ± **SD**	366.33 ± 110.24	394.00 ± 104.72	406.56 ± 114.12	396.23 ± 109.78	<0.001

^§^For % GT and FRC_CT_% analysis, data were available for 936 subjects.

^‡^For PRM data analysis, data were available for 932 subjects.

^#^For 6-MWT data analysis, data were available for 1,121 subjects.

BDR = bronchodilator response; BMI = body mass index; CAD = coronary artery disease; CHF = congestive heart failure; DM = diabetes mellitus; FRC_CT_% = functional residual capacity % predicted; HTN = hypertension; ICS = inhaled glucocorticosteroids, LABA = long-acting beta-agonist, LAMA = long-acting muscarinic antagonist, mMRC = modified Medical Research Council dyspnea score; OSA = obstructive sleep apnea; post-FEV1% = post-bronchodilator FEV1% predicted; post-FVC% = post-bronchodilator FVC% predicted; PRM^fSAD^ = parametric response mapping functional small airways disease; SD = standard deviation; SGRQ = St. George’s Respiratory Questionnaire score; TLC_CT_% = total lung capacity % predicted and 6-MWD = 6-min walk test.

**Figure 1:**
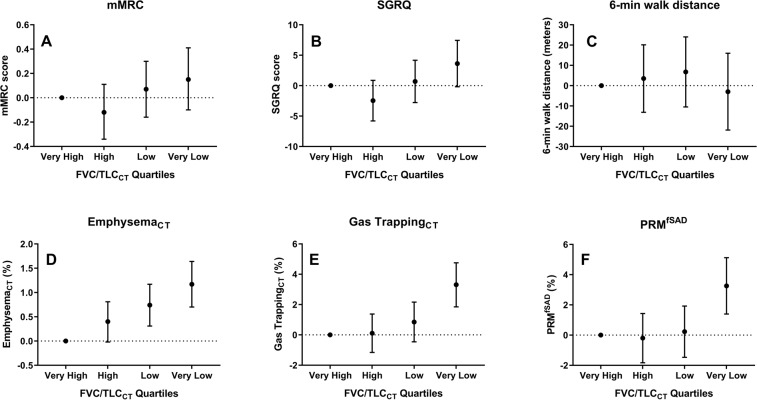
Associations between post-bronchodilator forced vital capacity/total lung capacity (FVC/TLC_CT_) quartiles at enrollment with dyspnea and health-related quality of life scores, chest CT % emphysema and % gas trapping, functional small airway disease, and 6-min walk test distance at baseline among smokers with Preserved Ratio Impaired Spirometry (PRISm; n = 1,131). Each panel in the figure represents a separate linear regression model with categorical post-bronchodilator FVC/TLC_CT_ quartile as the main independent variable (exposure) with the “very high” quartile used as the reference category. The dependent variable (outcome) in each model was (**A**) modified Medical Research Council (mMRC) dyspnea score, (**B**) St. George’s Respiratory Questionnaire total score (SGRQ), (**C**) 6-minute walk test distance (6-MWT in meters), (**D**) % Emphysema, (**E**) % Gas trapping, and (**F**) functional small airways disease (PRM^fSAD^). All models were adjusted for the following co-variates: age, sex, race, smoking status, smoking pack-years, body mass index (BMI), history of asthma and congestive heart failure, and diabetes mellitus. FVC/TLC_CT_ quartile is plotted on the x-axis while the regression coefficient (and 95% CI) for each category is plotted on the y-axis. ^*^For % GT analysis, n = 936 subjects. ^†^For PRM^fsad^ data analysis, n = 932 subjects. ^#^For 6-min walk test distance analysis, n = 1,121 subjects.

In multivariable-adjusted analyses, the very low FVC/TLC_CT_ quartile was associated with an average of 3.31% higher radiographic gas trapping (95% CI = 1.85–4.76; p < 0.001), and 3.26% higher PRM^fSAD^ (95% CI = 1.40–5.12; p < 0.001) relative to the very high quartile (Fig. 1). Lower quartiles were also associated with higher % emphysema. The very low quartile was associated with a trend towards higher SGRQ (3.63; 95% CI = −0.17 to 7.44; p = 0.06) (Supplementary Table S1).

### Progression to COPD at 5-year follow-up

**Figure 2:**
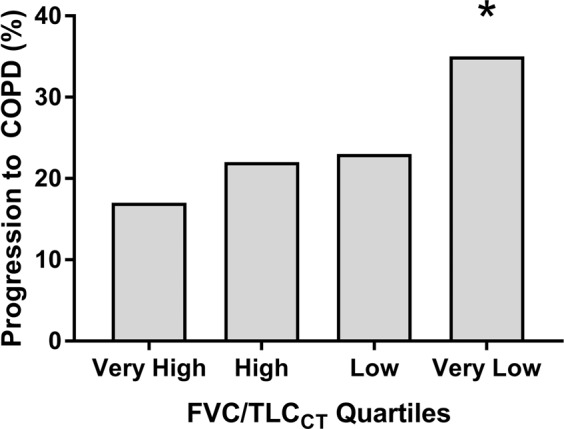
Progression to COPD (FEV_1_/FVC < 0.7) at the 5-year follow-up visit by post-bronchodilator forced vital capacity/total lung capacity ratio (FVC/TLC_CT_) quartiles at enrollment in smokers with preserved ratio impaired spirometry (n = 617). Cochran Armitage Trend test p < 0.001. Pairwise comparisons between quartiles performed using Chi-squared test. *p = 0.026 vs Very High FVC/TLC_CT_ Quartile.

Among participants with valid spirometry at the 5-year follow up visit (n = 617), approximately 35.9% (56 of 156) of individuals in very low FVC/TLC_CT_ quartile progressed to COPD, while 23% (37 of 160), 22% (35 of 156), and 17% (25 of 145) of individuals in the low, high, and very high FVC/TLC_CT_ quartiles, respectively, progressed to COPD (Fig. 2; Cochran-Armitage test for trend p < 0.001). In the multivariable-adjusted analysis, the very low FVC/TLC_CT_ quartile at enrollment was associated with progression to COPD with an OR of 2.67 (95% CI = 1.45–5.00; p < 0.001) relative to the highest quartile (Supplementary Table S2).

### Longitudinal changes in spirometry, functional capacity, and radiographic features

Supplement Table S2 shows changes in spirometry, functional capacity, and radiographic features between enrollment and follow-up visit. In the adjusted analysis, the very low FVC/TLC_CT_ quartile at enrollment was associated with increase of 2.74% radiographic gas trapping (95% CI = 0.55–4.93; p = 0.014) relative to the highest quartile (Supplementary Table S3). FVC/TLC_CT_ was not associated with change in FEV1, 6-MWT distance or % emphysema over time. There were no differences in the rate of decline in FEV1 by current smoking status at enrollment (combined and by FVC/TLC quartile - data not shown).

### Respiratory exacerbations

**Figure 3:**
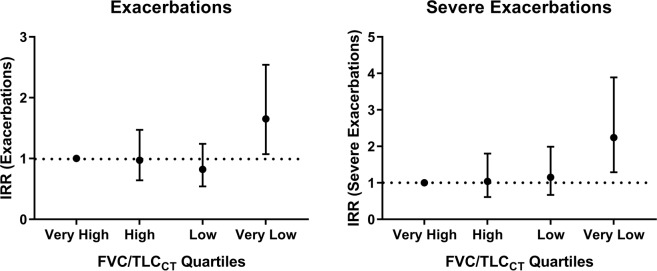
Associations between post-bronchodilator forced vital capacity/total lung capacity (FVC/TLC_CT_) quartiles at enrollment with prospective exacerbations and severe exacerbations in smokers with Preserved Ratio Impaired Spirometry (PRISm; n = 967). For exacerbation analysis, data for 967 subjects with PRISm at enrollment were available. Zero-inflated negative binomial regression models with post-bronchodilator FVC/TLC_CT_ as independent variable (exposure) and total exacerbations and severe exacerbations as the dependent variables (outcome) were performed. All regression models included the following co-variates: age, sex, race, body mass index, smoking status at the enrollment, smoking pack-years, history of asthma and congestive heart failure, and chronic bronchitis in the count negative binomial regression and an intercept-only model in the zero component. Follow-up time was included as an offset in the models. FVC/TLC_CT_ quartile is plotted on the x-axis while the IRR (and 95% CI) for each category is plotted on the y-axis. IRR = incidence rate ratio, FVC/TLC_CT_ = forced vital capacity/total lung capacity.

Of 967 subjects with exacerbation data available, 349 (36.1%) reported at least one exacerbation and 196 (20.3%) reported at least one severe exacerbation during a median follow-up time of 6.4 years (IQR = 3.8 to 7.4). Approximately, 44% (115 of 262), 37% (93 of 250), 31% (72 of 232), and 31% (69 of 223) in the very low, low, high, and very high Quartiles had at least one exacerbation during the time period (Cochran-Armitage trend test p < 0.001). In the very low, low, high, and very high Quartiles, 26% (67 of 262), 18% (46 of 250), 16% (38 of 232), and 20% (45 of 223) of participants, respectively, had at least one severe respiratory exacerbation, with a trend towards significance (Cochran-Armitage p = 0.095). We created multivariable zero-inflated negative binomial models to examine the association of FVC/TLC_CT_ quartile with respiratory exacerbations (Fig. 3). The very low FVC/TLC_CT_ quartile was associated with increased relative risk for total exacerbations (IRR = 1.65; 95% CI = 1.07–2.54; p = 0.023) and severe (IRR = 2.24; 95% CI = 1.29–3.89; p = 0.004) exacerbations relative to the “very high” FVC/TLC quartile (Supplementary Table S4).

### Mortality (n = 960)

**Figure 4:**
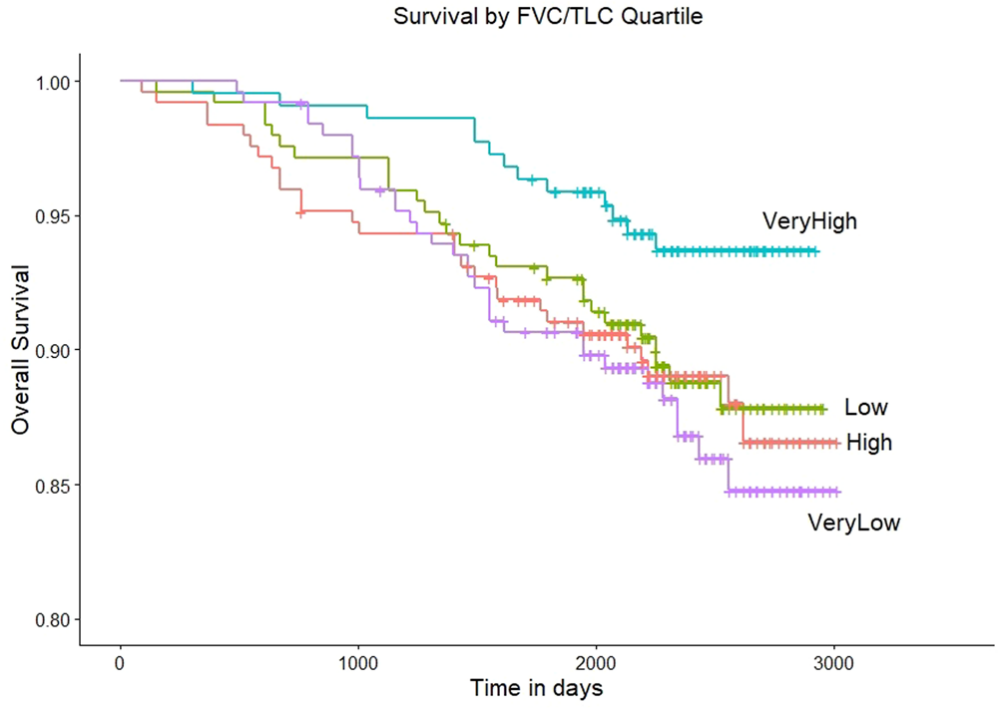
Kaplan-Meier Plot of overall survival by forced vital capacity/total lung capacity ratio (FVC/TLC_CT_) quartiles at enrollment in smokers with Preserved Ratio Impaired Spirometry (PRISm, n = 960). Chi-squared p-value for differences in mortality by quartile = 0.07.

During a median follow-up time of 2,408 days (IQR = 2158 to 2622), 12.9% (32 of 248) subjects died in the Very Low quartile, 11% (27 of 246) died in the low quartile, 11.3% (28 of 247) died in the High quartile, and 5.9% (13 of 219) died in the very high quartile (Cochran-Armitage trend test p = 0.02). A Kaplan-Meier plot of mortality by FVC/TLC_CT_ quartile at enrollment is shown in Fig. 4.

In Cox proportional hazards models adjusted for age at enrollment, sex, race, BMI, current smoking at enrollment, cumulative smoking exposure, diabetes, history of asthma and congestive heart failure, increased mortality in the high quartile with a trends towards an increased mortality in the low and very low quartiles relative to the very high quartile was observed (Table 2). In a Cox proportional hazards model examining individuals in the very high quartile relative to all other quartiles (high, low, very low) combined, a reduced risk of mortality was observed (HR = 0.53, 95% CI = 0.28–0.97,p = 0.040).

**Table 2:** Associations of post-bronchodilator forced vital capacity/total lung capacity (FVC/TLC_CT_) quartiles at enrollment with mortality in smokers with Preserved Ratio Impaired Spirometry (PRISm; n = 960).

Quartile	FVC/TLC_CT_			
	HR	2.5%	9.75%	P value
**Very High**	ref	ref	ref	ref
**High**	2.12	1.09	4.13	0.028
**Low**	1.68	0.84	3.36	0.14
**Very Low**	1.87	0.89	3.87	0.10

Cox Hazard regression models with post-bronchodilator FVC/TLCCT quartiles as independent variables (exposure) and mortality as the dependent variable(outcome) were performed.

All models for mortality included the following co-variates: age, sex, race, smoking status, smoking pack-years, body mass index (BMI), history of asthma and congestive heart failure, and diabetes mellitus.

HR = Hazard Ratio.

### Sensitivity analysis

When PRISm was defined using LLN criteria, we observed similar findings with those in main analysis except that FVC/TLC_CT_ was significantly associated with chronic bronchitis, increased mMRC and SGRQ and the association with mortality was attenuated (Supplementary Tables S5–S9 and Fig. S1**)**.

## Discussion

Our study explores the utility of FVC/TLC_CT_ ratio in former and current smokers with PRISm as a potential tool to identify individuals with features of and possible increased risk for progression to obstructive lung disease. In our cohort, very low FVC/TLC_CT_ was associated with radiographic findings traditionally associated with COPD as well as progression to COPD and respiratory exacerbations while very high FVC/TLC_CT_ was associated with reduced mortality.

PRISm is a common spirometric pattern with a prevalence between 5% and 20%^22–25^. Although often referred to as a “restrictive spirometric pattern”, 30–40% of patients with PRISm do not have reduced TLC^26,27^. On average, individuals PRISm have higher BMI, but obesity alone does not decrease vital capacity or TLC below the LLN in most individuals^28^. Notably, only about 5% of patients undergoing bariatric surgery for extreme obesity have PRISm at preoperative assessment^29^.

PRISm is comprised of a heterogeneous population with a wide range of BMI, degree of lung function impairment, and radiographic emphysema likely due to different underlying pathological processes in each individual^2^. Subgroups within PRISm may have increased risk for FEV_1_ decline, progression to COPD, exacerbations, and mortality. In this manuscript, we utilize FVC/TLC, which decreases in obstructive lung disease^11^, as a conceptual surrogate for RV (which was not directly measured in our cohort) to identify individuals with features obstructive lung disease within PRISm. Our finding that individuals with PRISm with low FVC/TLC have increased radiographic emphysema and gas trapping complements work from the SPIROMICS cohort, where that RV/TLC was shown to be associated with increased radiographic emphysema and gas trapping in smokers with normal lung function^30^. Apart from the fact that RV/TLC was not available in our cohort, we used FVC/TLC as it may be more sensitive to identify the presence of small airway disease than RV/TLC because FVC, a dynamic measure obtained at forced expiration, will capture dynamic collapse and air trapping not present during slow exhalation maneuver^31,32^. Future studies should examine the role of RV/TLC in PRISm. In addition, we did not examine FRC/TLC as FRC can be reduced remarkably in obesity^28^ which may render difficult to interpret those measures when an obstructive lung diseases coexists.

Our findings suggest that low FVC/TLC_CT_ may be a possible a marker of early obstructive pulmonary disease. Nevertheless, participants in the very low FVC/TLC_CT_ quartile have higher BMI; this contrasts with the common knowledge that patients with established obstructive pulmonary disease have often lower BMI. Previous studies have shown an inverse relationship of BMI with mortality in COPD, known also as the “obesity paradox” with confounders such as exercise capacity and muscle mass possibly contributing towards favorable outcomes^33,34^. In addition, despite the fact that obesity does not typically reduce the FVC below the LLN in subjects without lung disease^29^, higher BMI decreases FVC and increases the FEV_1_/FVC ratio which can lead to underdiagnosis of obstructive pulmonary disease^31,35^. In COPD subjects with established airflow obstruction, increasing BMI is associated with higher FEV_1_/FVC^35^.

We acknowledge that the fixed threshold FEV1/FVC < 0.7 diagnostic criterion for COPD endorsed by Global Initiative for Chronic Obstructive Lung Disease(GOLD) may have also misclassified individuals with obstructive lung disease as PRISm. In a recent large population-based sample (n = 24,207), Bhatt and colleagues showed that the discriminative accuracy of FEV_1_/FVC < 0.7 to predict COPD-related death and/or hospitalization was not inferior to FEV_1_/FVC < LLN^36^. We assert that because the majority of our findings remained robust on sensitivity analyses using LLN-defined lung function categories. FVC/TLC ratio can be utilized to identify individuals with features of obstructive lung disease regardless of whether fixed-threshold or LLN criteria are used.

In COPDGene, 40.5% of PRISm individuals and 32.5% of smokers with normal lung function are African American^2^. Differences in the reliability of prediction equations may lead to the “overdiagnosis” of African American with PRISm in the absence of true pathology; this may contribute to the lower rates of African Americans in the low FVC/TLC_CT_ quartiles. It is also unclear why females were relatively over-represented in the lower FVC/TLC_CT_ quartiles. Whether PRISm represents a gender-specific pathway to COPD, or whether traditional FEV_1_/FVC criteria systematically misclassify women with COPD is not known^37,38^. Our sensitivity analysis using gender and race specific spirometric criteria to define PRISm showed similar findings. Future studies, especially in cohorts of diverse ancestry and ethnicity, are warranted to further explore these findings.

Previous studies have shown that air trapping is associated with FEV_1_ decline. In current and former smokers with at least 20 pack-years smoking and normal lung function, RV/TLC is associated with FEV_1_ decline^30^. We have extended these finding by showing that air trapping (low FVC/TLC) in individuals with PRISm is associated with progression to COPD. General population studies have also shown that individuals with abnormal non-obstructed lung function are at risk for developing COPD^39,40^. It may seem counterintuitive that low FVC/TLC_CT_ in PRISm was associated with progression to COPD and respiratory exacerbations, but was not associated with FEV_1_ decline, increase in emphysema, and change in 6-MWT distance over time^41^. Within COPDGene, individuals with PRISm are at increased risk for respiratory exacerbations relative to smokers with normal lung function^42^. However, respiratory exacerbations in PRISm do not result in significant excess lung function decline in FEV_1_ as observed in individuals with established airflow limitation^43^. A survivor bias may also be present in FEV1 decline analysis^43^. Participants that had poor lung function and low FVC/TLC may have died before the follow-up visit. Similarly, we may have not observed changes in 6-MWT distance likely due to the high variability of the test^44,45^.

Population-based studies have shown that PRISm is associated with increased cardiac^7^ and all-cause mortality^1,22^. While the increased average BMI in PRISm as a whole may mediate some of the risk associated with increased mortality, the association between very high FVC/TLC_CT_ and lower mortality relative to all other quartiles despite concurrent adjustment for BMI, congestive heart failure, and diabetes status in our study suggests our composite measure may have utility in the risk-stratification of individuals with PRISm. Previous studies in COPD have shown associations between RV and mortality^46^. In Veterans with a history of smoking and normal lung function, Zeng *et al*. showed that RV/TLC is associated with increased respiratory medication use, hospitalizations, and all-cause mortality^47^.

Our study is the first one showing that a composite measure of lung function may help to identify patients with PRISm who eventually progress to classic airflow obstruction and are at increased risk for respiratory exacerbations and death. The strengths of our study include large sample size, highly granular epidemiological data, axial radiographic imaging data, and longitudinal data on clinically relevant outcomes. Despite this, we acknowledge the following limitations. RV was not available in our cohort. TLC_CT_ was measured in supine position by chest CT, which is usually lower than TLC measured in seated position by plethysmography^13^. Another limitation is possible self-selection bias of subjects who returned for a follow-up visit. Since our cohort includes only smokers, our findings cannot be generalized to non-smoking populations and future studies in independent cohorts are warranted. In conclusion, FVC/TLC_CT_ can help to identify individuals with PRISm at increased risk for clinical events and progression to COPD, and who would benefit from smoking cessation and may be a potential target population for treatment trials in the future.

### Grant support and disclaimer

The project described was supported by Award Number U01 HL089897 and Award Number U01 HL089856 from the National Heart, Lung, and Blood Institute. The content is solely the responsibility of the authors and does not necessarily represent the official views of the National Heart, Lung, and Blood Institute or the National Institutes of Health. Department of Veterans Affairs, Veterans Health Administration, Office of Rural Health, Veterans Rural Health Resource Center (Award # 14380), and the Health Services Research and Development (HSR&D) Service through the Comprehensive Access and Delivery Research and Evaluation (CADRE) Center (CIN 13-412)U.S. Department of Veterans Affairs IK2RX002165. The views expressed in this article are those of the authors and do not necessarily reflect the position or policy of the Department of Veterans Affairs or the United States Government.

### COPD foundation funding

The COPDGene project is also supported by the COPD Foundation through contributions made to an Industry Advisory Board comprised of AstraZeneca, Boehringer Ingelheim, GlaxoSmithKline, Novartis, Pfizer, Siemens and Sunovion.

### COPDGene Investigators – core units

*Administrative Center*: James D. Crapo, MD (PI); Edwin K. Silverman, MD, PhD (PI); Barry J. Make, MD; Elizabeth A. Regan, MD, PhD.

*Genetic Analysis Center*: Terri Beaty, PhD; Ferdouse Begum, PhD; Peter J. Castaldi, MD, MSc; Michael Cho, MD; Dawn L. DeMeo, MD, MPH; Adel R. Boueiz, MD; Marilyn G. Foreman, MD, MS; Eitan Halper-Stromberg; Lystra P. Hayden, MD, MMSc; Craig P. Hersh, MD, MPH; Jacqueline Hetmanski, MS, MPH; Brian D. Hobbs, MD; John E. Hokanson, MPH, PhD; Nan Laird, PhD; Christoph Lange, PhD; Sharon M. Lutz, PhD; Merry-Lynn McDonald, PhD; Margaret M. Parker, PhD; Dandi Qiao, PhD; Elizabeth A. Regan, MD, PhD; Edwin K. Silverman, MD, PhD; Emily S. Wan, MD; Sungho Won, Ph.D.; Phuwanat Sakornsakolpat, M.D.; Dmitry Prokopenko, Ph.D.

*Imaging Center*: Mustafa Al Qaisi, MD; Harvey O. Coxson, PhD; Teresa Gray; MeiLan K. Han, MD, MS; Eric A. Hoffman, PhD; Stephen Humphries, PhD; Francine L. Jacobson, MD, MPH; Philip F. Judy, PhD; Ella A. Kazerooni, MD; Alex Kluiber; David A. Lynch, MB; John D. Newell, Jr., MD; Elizabeth A. Regan, MD, PhD; James C. Ross, PhD; Raul San Jose Estepar, PhD; Joyce Schroeder, MD; Jered Sieren; Douglas Stinson; Berend C. Stoel, PhD; Juerg Tschirren, PhD; Edwin Van Beek, MD, PhD; Bram van Ginneken, PhD; Eva van Rikxoort, PhD; George Washko, MD; Carla G. Wilson, MS; PFT QA Center, Salt Lake City, UT: Robert Jensen, PhD.

*Data Coordinating Center and Biostatistics*, *National Jewish Health, Denver, CO*: Douglas Everett, PhD; Jim Crooks, PhD; Camille Moore, PhD; Matt Strand, PhD; Carla G. Wilson, MS.

*Epidemiology Core*, *University of Colorado Anschutz Medical Campus, Aurora, CO*: John E. Hokanson, MPH, PhD; John Hughes, PhD; Gregory Kinney, MPH, PhD; Sharon M. Lutz, PhD; Katherine Pratte, MSPH; Kendra A. Young, PhD.

*Mortality Adjudication Core:* Surya Bhatt, MD; Jessica Bon, MD; MeiLan K. Han, MD, MS; Barry Make, MD; Carlos Martinez, MD, MS; Susan Murray, ScD; Elizabeth Regan, MD; Xavier Soler, MD; Carla G. Wilson, MS.

*Biomarker Core*: Russell P. Bowler, MD, PhD; Katerina Kechris, PhD; Farnoush Banaei-Kashani, Ph.D.

### COPDGene Investigators – clinical centers

*Ann Arbor VA:* Jeffrey L. Curtis, MD; Carlos H. Martinez, MD, MPH; Perry G. Pernicano, MD.

*Baylor College of Medicine, Houston, TX*: Nicola Hanania, MD, MS; Philip Alapat, MD; Mustafa Atik, MD; Venkata Bandi, MD; Aladin Boriek, PhD; Kalpatha Guntupalli, MD; Elizabeth Guy, MD; Arun Nachiappan, MD; Amit Parulekar, MD;

*Brigham and Women’s Hospital, Boston, MA*: Dawn L. DeMeo, MD, MPH; Craig Hersh, MD, MPH; Francine L. Jacobson, MD, MPH; George Washko, MD.

*Columbia University, New York, NY*: R. Graham Barr, MD, DrPH; John Austin, MD; Belinda D’Souza, MD; Gregory D.N. Pearson, MD; Anna Rozenshtein, MD, MPH, FACR; Byron Thomashow, MD.

*Duke University Medical Center, Durham, NC*: Neil MacIntyre, Jr., MD; H. Page McAdams, MD; Lacey Washington, MD.

*HealthPartners Research Institute, Minneapolis, MN*: Charlene McEvoy, MD, MPH; Joseph Tashjian, MD.

*Johns Hopkins University, Baltimore, MD*: Robert Wise, MD; Robert Brown, MD; Nadia N. Hansel, MD, MPH; Karen Horton, MD; Allison Lambert, MD, MHS; Nirupama Putcha, MD, MHS.

*Los Angeles Biomedical Research Institute at Harbor UCLA Medical Center, Torrance, CA*: Richard Casaburi, PhD, MD; Alessandra Adami, PhD; Matthew Budoff, MD; Hans Fischer, MD; Janos Porszasz, MD, PhD; Harry Rossiter, PhD; William Stringer, MD.

*Michael E. DeBakey VAMC, Houston*, *TX*: Amir Sharafkhaneh, MD, PhD; Charlie Lan, DO.

*Minneapolis VA:* Christine Wendt, MD; Brian Bell, MD.

*Morehouse School of Medicine, Atlanta, GA*: Marilyn G. Foreman, MD, MS; Eugene Berkowitz, MD, PhD; Gloria Westney, MD, MS

*National Jewish Health, Denver, CO*: Russell Bowler, MD, PhD; David A. Lynch, MB.

*Reliant Medical Group, Worcester, MA*: Richard Rosiello, MD; David Pace, MD.

*Temple University, Philadelphia, PA:* Gerard Criner, MD; David Ciccolella, MD; Francis Cordova, MD; Chandra Dass, MD; Gilbert D’Alonzo, DO; Parag Desai, MD; Michael Jacobs, PharmD; Steven Kelsen, MD, PhD; Victor Kim, MD; A. James Mamary, MD; Nathaniel Marchetti, DO; Aditi Satti, MD; Kartik Shenoy, MD; Robert M. Steiner, MD; Alex Swift, MD; Irene Swift, MD; Maria Elena Vega-Sanchez, MD.

*University of Alabama, Birmingham, AL:* Mark Dransfield, MD; William Bailey, MD; Surya Bhatt, MD; Anand Iyer, MD; Hrudaya Nath, MD; J. Michael Wells, MD.

*University of California, San Diego, CA*: Joe Ramsdell, MD; Paul Friedman, MD; Xavier Soler, MD, PhD; Andrew Yen, MD.

*University of Iowa, Iowa City, IA*: Alejandro P. Comellas, MD; Karin F. Hoth, PhD; John Newell, Jr., MD; Brad Thompson, MD.

*University of Michigan, Ann Arbor, MI*: MeiLan K. Han, MD, MS; Ella Kazerooni, MD; Carlos H. Martinez, MD, MPH.

*University of Minnesota, Minneapolis, MN*: Joanne Billings, MD; Abbie Begnaud, MD; Tadashi Allen, MD.

*University of Pittsburgh, Pittsburgh, PA*: Frank Sciurba, MD; Jessica Bon, MD; Divay Chandra, MD, MSc; Carl Fuhrman, MD; Joel Weissfeld, MD, MPH.

*University of Texas Health Science Center at San Antonio, San Antonio, TX*: Antonio Anzueto, MD; Sandra Adams, MD; Diego Maselli-Caceres, MD; Mario E. Ruiz, MD.

**Table:** 

Clinical Center	Institution Title	Protocol Number
National Jewish Health	National Jewish IRB	HS-1883a
Brigham and Women’s Hospital	Partners Human Research Committee	2007-P-000554/2; BWH
Baylor College of Medicine	Institutional Review Board for Baylor College of Medicine and Affiliated Hospitals	H-22209
Michael E. DeBakey VAMC	Institutional Review Board for Baylor College of Medicine and Affiliated Hospitals	H-22202
Columbia University Medical Center	Columbia University Medical Center IRB	IRB-AAAC9324
Duke University Medical Center	The Duke University Health System Institutional Review Board for Clinical Investigations (DUHS IRB)	Pro00004464
Johns Hopkins University	Johns Hopkins Medicine Institutional Review Boards (JHM IRB)	NA_00011524
Los Angeles Biomedical Research Institute	The John F. Wolf, MD Human Subjects Committee of Harbor-UCLA Medical Center	12756–01
Morehouse School of Medicine	Morehouse School of Medicine Institutional Review Board	07–1029
Temple University	Temple University Office for Human Subjects Protections Institutional Review Board	11369
University of Alabama at Birmingham	The University of Alabama at Birmingham Institutional Review Board for Human Use	FO70712014
University of California, San Diego	University of California, San Diego Human Research Protections Program	070876
University of Iowa	The University of Iowa Human Subjects Office	200710717
Ann Arbor VA	VA Ann Arbor Healthcare System IRB	PCC 2008-110732
University of Minnesota	University of Minnesota Research Subjects’ Protection Programs (RSPP)	0801M24949
University of Pittsburgh	University of Pittsburgh Institutional Review Board	PRO07120059
University of Texas Health Sciences Center at San Antonio	UT Health Science Center San Antonio Institutional Review Board	HSC20070644H
Health Partners Research Foundation	Health Partners Research Foundation Institutional Review Board	07–127
University of Michigan	Medical School Institutional Review Board (IRBMED)	HUM00014973
Minneapolis VA Medical Center	Minneapolis VAMC IRB	4128-A
Fallon Clinic	Institutional Review Board/Research Review Committee Saint Vincent Hospital – Fallon Clinic – Fallon Community Health Plan	1143

## Supplementary Information

Supplementary Information.
