# Supplementary material for: Low FVC/TLC in Preserved Ratio Impaired Spirometry (PRISm) is associated with features of and progression to obstructive lung disease
Source: Sci Rep. 2020 Mar 20;10:5169. doi: 10.1038/s41598-020-61932-0 (PMC7083974; doi:10.1038/s41598-020-61932-0)
Supplement: Supplementary file 1 — Supplementary Information. [file 41598_2020_61932_MOESM1_ESM.docx]

**Supplementary material**

**Low FVC/TLC in Preserved Ratio Impaired Spirometry (PRISm) is associated with features of and progression to obstructive lung disease**

Spyridon Fortis^1,2^

Alejandro Comellas^1^

Victor Kim^3^

Richard Casaburi^4^

John Hokanson^5^

James Crapo^6^

Edwin K. Silverman^7^

Emily S. Wan^7,8^

^1^ Division of Pulmonary, Critical Care and Occupational Medicine, University of Iowa Hospital and Clinics, Iowa City, IA, USA.

^2^ Center for Access & Delivery Research & Evaluation (CADRE), Iowa City VA Health Care System, Iowa City, IA, USA.

^3^ Division of Pulmonary and Critical Care Medicine, Department of Medicine, Temple University School of Medicine, Philadelphia, PA, USA.

^4^ Los Angeles Biomedical Research Institute at Harbor-UCLA Medical Center, Torrance, CA, USA.

^5^ Department of Epidemiology, Colorado School of Public Health, University of Colorado Denver, Denver, CO.

^6^Department of Medicine, National Jewish Health, Denver, CO,USA.

^7^ Channing Division of Network Medicine, Brigham and Women's Hospital, Boston, MA, USA.

^8^ VA Boston Healthcare System, Jamaica Plain, MA, USA

**Supplementary Table S1.** Associations of post-bronchodilator forced vital capacity /total lung capacity (FVC/TLC_CT_) quartiles at enrollment with chronic bronchitis, dyspnea and health-related quality of life scores, chest CT % emphysema and % gas trapping, functional small airway disease, and 6-min walk test distance in smokers with preserved ratio impaired spirometry (n=1,131).

|  | **FVC/TLC_CT_** | | | | |
| --- | --- | --- | --- | --- | --- |
| **Chronic Bronchitis** | OR | 2.5% | 97.5% | P value | |
| **Very High** | ref | ref | ref | ref | |
| **High** | 1.49 | 0.94 | 2.38 | 0.09 | |
| **Low** | 1.36 | 0.84 | 2.20 | 0.21 | |
| **Very Low** | 1.43 | 0.85 | 2.42 | 0.18 | |
| **mMRC** | coef | 2.5% | 97.5% | P value |  |
| **Very High** | ref | ref | ref | ref |  |
| **High** | -0.12 | -0.34 | 0.11 | 0.30 |  |
| **Low** | 0.07 | -0.16 | 0.30 | 0.55 |  |
| **Very Low** | 0.15 | -0.10 | 0.41 | 0.24 |  |
| **SGRQ** | coef | 2.5% | 97.5% | P value | |
| **Very High** | ref | ref | ref | ref | |
| **High** | -2.46 | -5.80 | 0.88 | 0.15 | |
| **Low** | 0.69 | -2.78 | 4.17 | 0.70 | |
| **Very Low** | 3.63 | -0.17 | 7.44 | 0.06 | |
| **% Emphysema** | coef | 2.5% | 97.5% | P value | |
| **Very High** | ref | ref | ref | ref | |
| **High** | 0.40 | -0.02 | 0.81 | 0.06 | |
| **Low** | 0.74 | 0.31 | 1.17 | <0.001 | |
| **Very Low** | 1.17 | 0.70 | 1.64 | <0.001 | |
| *******% Gas trapping** | coef | 2.5% | 97.5% | P value | |
| **Very High** | ref | ref | ref | ref | |
| **High** | 0.11 | -1.16 | 1.38 | 0.86 | |
| **Low** | 0.85 | -0.46 | 2.16 | 0.20 | |
| **Very Low** | 3.31 | 1.85 | 4.76 | <0.001 | |
| **^†^** **%PRM^fSAD^** | coef | 2.5% | 97.5% | P value | |
| **Very High** | ref | ref | ref | ref | |
| **High** | -0.20 | -1.83 | 1.43 | 0.81 | |
| **Low** | 0.23 | -1.47 | 1.92 | 0.79 | |
| **Very Low** | 3.26 | 1.40 | 5.12 | <0.001 | |
| **^#^6-MWT distance, meters** | coef | 2.5% | 97.5% | P value | |
| **Very High** | ref | ref | ref | ref | |
| **High** | 3.50 | -13.15 | 20.15 | 0.68 | |
| **Low** | 6.79 | -10.46 | 24.03 | 0.44 | |
| **Very Low** | -2.96 | -21.90 | 15.97 | 0.76 | |

Binary logistic regression models with post-bronchodilator FVC/TLC_CT_ quartiles as independent variables (exposure) and chronic bronchitis as the dependent variables (outcome) were performed. Linear regression models with post-bronchodilator FVC/TLC_CT_ quartiles as independent variables (exposure) and mMRC, SGRQ, % Emphysema, % Gas trapping, PRM^fSAD^, and 6-MWT distance as the dependent variables (outcome) were performed. All models included the following co-variates: age, sex, race, body mass index, smoking status at the enrollment, smoking pack-years, history of asthma and congestive heart failure.

* For % GT analysis, data were available for 936 participants.

† For PRM^fSAD^data analysis, data were available for 932 participants.

#For 6-MWT distance analysis, data were available for 1,121 participants.

mMRC = modified Medical Research Council dyspnea score; OR=odds ratio; PRM^fSAD^= parametric response mapping functional small airways disease; SGRQ = St. George’s Respiratory Questionnaire score; 6-MWT = 6-min walk test.

**Supplementary Table S2.** Change in FEV_1_, 6-MWT distance, % emphysema and gas trapping between enrollment and follow-up, and progression to COPD at 5-year follow-up visit in smokers with Preserved Ratio Impaired Spirometry (PRISm) across post-bronchodilator forced vital capacity /total lung capacity ratio (FVC/TLC_CT_) quartiles (n=617).

|  | **Very Low quartile**  **(n=156)** | **Low quartile**  **(n=160)** | **High quartile**  **(n=156)** | **Very High quartile**  **(n=145)** | **P for trend** |
| --- | --- | --- | --- | --- | --- |
| **FVC/TLC_CT_** | **<0.53** | **0.53 - 0.59** | **0.59-0.66** | **>0.66** |  |
| **Change in FEV1 (ml/yr)** | -18.26 ± 53.68 | -18.03 ± 49.98 | -26.15 ± 47.94 | -18.27 ± 55.49 | 0.17 |
| **Change in 6-MWT (meters/year)*** | -43.0 ± 94.03 | -41.13 ± 101.75 | -37.07 ± 117.17 | -34.83± 105.47 | 0.066 |
| **Change in %Emph per year^§^** | 0.06 ± 2.03 | -0.18 ± 1.79 | -0.12 ± 2.01 | 0.07 ± 1.71 | 0.63 |
| **Change in %GT per year** ‡ | 2.07 ± 6.58 | 0.11 ± 6.02 | 0.01 ± 6.52 | -0.03 ± 5.46 | 0.073 |
| **COPD at follow-up visit** | 56 (38.9%) | 37 (23.1%) | 35 (22.4%) | 25 (17.2%) | <0.001 |

* For Change in 6-MWT analysis, data were available for 154,155,154, and 144 participants for very low, low, high, and very high quartile, respectively.

^§^ For % Emp analysis, data were available for 123,120,128, and 108 participants for very low, lo, high, and very high quartile, respectively.

‡ For Change in %GT analysis, data were available for 92, 95, 94, and 72 participants for very low, lo, high, and very high quartile, respectively.

**Supplementary Table S3.** Multivariable-adjusted associations between post-bronchodilator forced vital capacity /total lung capacity (FVC/TLC_CT_) quartiles at enrollment with change in FEV_1_, 6-MWT distance, % emphysema and gas trapping between enrollment and follow-up, and progression to COPD at 5-year follow-up visit in smokers with preserved ratio impaired spirometry(n=617).

|  | **FVC/TLC_CT_** | | | |
| --- | --- | --- | --- | --- |
| **Change in FEV1, ml/year** | coef | 2.5% | 9.75% | P value |
| **Very High** | ref | ref | ref | ref |
| **High** | -0.20 | -8.95 | 3.97 | 0.20 |
| **Low** | 3.85 | -7.85 | 15.55 | 0.52 |
| **Very Low** | 3.75 | -8.95 | 16.45 | 0.56 |
| **Change in 6-MWT distance, meters/year** | coef | 2.5% | 9.75% | P value |
| **Very High** | ref | ref | ref | ref |
| **High** | -0.54 | -24.47 | 23.40 | 0.96 |
| **Low** | -1.87 | -26.57 | 22.83 | 0.88 |
| **Very Low** | 4.09 | -22.55 | 30.73 | 0.76 |
| **Change in % Emphysema pe yer** | coef | 2.5% | 9.75% | P value |
| **Very High** | ref | ref | ref | ref |
| **High** | -0.08 | -0.58 | 0.41 | 0.75 |
| **Low** | -0.07 | -0.59 | 0.45 | 0.79 |
| **Very Low** | 0.19 | -0.37 | 0.75 | 0.51 |
| **Change in % Gas trapping per year** | coef | 2.5% | 9.75% | P value |
| **Very High** | ref | ref | ref | ref |
| **High** | 0.32 | -1.60 | 2.24 | 0.74 |
| **Low** | 1.02 | -0.96 | 3.00 | 0.31 |
| **Very Low** | 2.74 | 0.55 | 4.93 | 0.014 |
| **COPD at 5 years** | OR | 2.5% | 9.75% | P value |
| **Very High** | ref | ref | ref | ref |
| **High** | 1.49 | 0.83 | 2.72 | 0.18 |
| **Low** | 1.54 | 0.85 | 2.83 | 0.16 |
| **Very Low** | 2.67 | 1.45 | 5.00 | 0.002 |

Linear regression models with post-bronchodilator FVC/ quartiles as independent variables (exposure) change in FEV1, 6-MWT distance, % emphysema and gas trapping between enrollment and follow-up as the dependent variables(outcomes) were performed. Binary logistic regression models with post-bronchodilator FVC/TLC_CT_ quartiles as independent variables (exposure) and progression to COPD at follow-up visit as the dependent variables (outcome) were performed. All models included the following co-variates: age, sex, race, body mass index, smoking status at the enrollment, smoking pack-years, history of asthma and congestive heart failure.

For change in FEV_1_ mL/year, data were available for 617 participants.

For change in 6-MWT distance analysis, data were available for 606 participants.

For change in % emphysema analysis, data were available for 478 participants.

For change in % gas trapping analysis, data were available for 352 participants.

OR= odds ratio; 6-MWT = 6-min walk test.

**Supplementary Table S4.** Multivariable-adjusted associations between post-bronchodilator forced vital capacity /total lung capacity (FVC/TLC_CT_) quartiles at enrollment with prospective total exacerbations and severe exacerbations in smokers with preserved ratio impaired spirometry (n=967).

|  | **FVC/TLC_CT_** | | | |
| --- | --- | --- | --- | --- |
| **Exacerbations** | IRR | 2.5% | 9.75% | P value |
| **Very High** | ref | ref | ref | ref |
| **High** | 0.97 | 0.64 | 1.47 | 0.88 |
| **Low** | 0.82 | 0.54 | 1.24 | 0.34 |
| **Very low** | 1.65 | 1.07 | 2.54 | 0.023 |
| **Severe Exacerbations** | IRR | 2.5% | 9.75% | P value |
| **Very High** | ref | ref | ref | ref |
| **High** | 1.04 | 0.61 | 1.80 | 0.88 |
| **Low** | 1.15 | 0.67 | 1.99 | 0.61 |
| **Very Low** | 2.24 | 1.29 | 3.89 | 0.004 |

For exacerbation analysis, data for 967 of total 1131participants were available. Zero-inflated negative binomial regression models with post-bronchodilator FVC/TLC_CT_ quartile as independent variables (exposure) and total exacerbations and severe exacerbations as the dependent variables (outcome) were performed. All regression models included the following co-variates: age, sex, race, body mass index, smoking status at the enrollment, smoking pack-years, history of asthma and congestive heart failure, and chronic bronchitis in the count negative binomial regression and an intercept-only model in the zero component. Follow-up time was included as an offset in the models.

IRR= incident rate ratio.

**Sensitivity analysis**

There were 1,096 participants with PRISm defined as post-bronchodilator FEV_1_/FVC≥ the lower limit of normal (LLN) and FEV1< LLN. After excluding 3 with bronchiectasis, 9 with interstitial lung disease, 106 with no available TLC, and 5 that FVC/TLC≥1, 973 participants were included in the analyses.

**Supplementary Table S5.** Baseline characteristics of smokers with preserved ratio impaired spirometry defined based on the lower limit of normal across post-bronchodilator forced vital capacity /total lung capacity ratio (FVC/TLC_CT_) quartiles (n=973).

|  | Very Low quartile  (n=244) | Low quartile  (n=243) | High quartile  (n=243) | Very High quartile  (n=243) | P for trend |
| --- | --- | --- | --- | --- | --- |
| FVC/TLC_CT_ | **<0.52** | **0.52 - 0.58** | **0.58-0.65** | **>0.65** |  |
| Age, y ± SD | 63.93 ± 8.39 | 58.81 ± 7.55 | 56.36 ± 7.41 | 53.27 ± 6.7 | <0.001 |
| Female, n(%) | 127 (52.0%) | 124 (51.0%) | 108 (44.4%) | 79 (32.5%) | <0.001 |
| African American, n(%) | 49 (20.1%) | 59 (24.3%) | 74 (30.5%) | 109 (44.9%) | <0.001 |
| BMI, Kg/m^2^ ± SD | 33.02 ± 7.13 | 33.64 ± 7.54 | 30.75 ± 6.93 | 31.14 ± 7.02 | <0.001 |
| Pack-Years ± SD | 52.53 ± 28.60 | 48.48 ± 24.93 | 44.69 ± 29.05 | 37.70 ± 20.20 | <0.001 |
| Active Smoker, n(%) | 123 (50.4%) | 141 (58.0%) | 145 (59.7%) | 170 (70.0%) | <0.001 |
| Chronic Bronchitis, n(%) | 56 (23.0%) | 46 (18.9%) | 58 (23.9%) | 29 (11.9%) | <0.001 |
| mMRC ± SD | 1.85 ± 1.48 | 1.47 ± 1.45 | 1.30 ± 1.40 | 1.30 ± 1.45 | <0.001 |
| SGRQ ± SD | 34.65 ± 21.94 | 31.03 ± 24.22 | 27.48 ± 22.40 | 28.25 ± 23.24 | <0.001 |
| Asthma, n(%) | 65 (26.6%) | 53 (21.8%) | 45 (18.5%) | 48 (19.8%) | 0.042 |
| CHF, n(%) | 23 (9.4%) | 13 (5.3%) | 8 (3.3%) | 8 (3.3%) | 0.001 |
| DM, n(%) | 71 (29.1%) | 72 (29.6%) | 51 (21.0%) | 35 (14.4%) | <0.001 |
| HTN, n(%) | 138 (56.6%) | 132 (54.3%) | 107 (44.0%) | 97 (39.9%) | <0.001 |
| CAD, n(%) | 36 (14.8%) | 31 (12.8%) | 21 (8.6%) | 10 (4.1%) | <0.001 |
| OSA, n(%) | 72 (29.5%) | 60 (24.7%) | 58 (23.9%) | 42 (17.3%) | 0.002 |
| CVA, n(%) | 10 (4.1%) | 13 (5.3%) | 7 (2.9%) | 5 (2.1%) | 0.11 |
| LAMA, n(%) | 35 (15%) | 19 (8.1%) | 17 (7.1%) | 14 (5.9%) | <0.001 |
| ICS, n(%) | 24 (10.1%) | 17 (7.2%) | 15 (6.3%) | 10 (4.2%) | 0.011 |
| LABA, n(%) | 11 (4.6%) | 2 (0.9%) | 2 (0.8%) | 5 (2.1%) | 0.067 |
| ICS/LABA, n(%) | 61 (25.7%) | 38 (16.0%) | 22 (9.2%) | 21 (8.7%) | <0.001 |
| Post-FEV1% ± SD | 61.20 ± 8.73 | 67.94 ± 7.00 | 69.79 ± 6.37 | 70.26 ± 6.21 | <0.001 |
| Post-FVC% ± SD | 64.03 ± 9.48 | 70.44 ± 7.47 | 72.27 ± 7.68 | 72.88 ± 7.20 | <0.001 |
| BDR, n(%) | 41 (17.1%) | 30 (12.5%) | 36 (15.0%) | 39 (16.1%) | 0.97 |
| ^§^% Emphysema ± SD | 2.78 ± 4.25 | 1.84 ± 2.39 | 1.73 ± 2.39 | 1.23 ± 1.79 | <0.001 |
| ^§^% Gas trapping ± SD | 15.90 ± 10.54 | 11.30 ± 9.00 | 9.46 ± 7.50 | 7.83 ± 6.36 | <0.001 |
| ^‡^PRM^fSAD^, % ± SD | 18.00 ± 11.23 | 13.07 ± 9.91 | 10.80 ± 8.26 | 10. 01 ± 8.39 | <0.001 |
| ^§^FRC_CT_% ± SD | 100.32 ± 19.07 | 90.28 ± 15.53 | 83.52 ± 14.70 | 75.78 ± 12.06 | <0.001 |
| TLC_CT_ % ± SD | 90.12 ± 13.22 | 85.64 ± 10.45 | 80.01 ± 10.65 | 69.81 ± 9.61 | <0.001 |
| ^#^6-MWT, meters ± SD | 366.65 ± 112.41 | 383.49 ± 104.38 | 413.74 ± 112.99 | 413.71 ± 112.13 | <0.001 |

^§^ For % GT and FRC_CT_% analysis, data were available for 819 participants.

‡ For PRM data analysis, data were available for 806 participants.

## ^#^ For 6-MWT data analysis, data were available for 962 participants.

BDR = bronchodilator response; BMI = body mass index; CAD = coronary artery disease; CHF = congestive heart failure; DM = diabetes mellitus; FRC_CT_% = functional residual capacity % predicted; HTN = hypertension; ICS = inhaled glucocorticosteroids, LABA = long-acting beta-agonist, LAMA = long-acting muscarinic antagonist, mMRC = modified Medical Research Council dyspnea score; OSA = obstructive sleep apnea; post-FEV1% = post-bronchodilator FEV1% predicted; post-FVC% = post-bronchodilator FVC% predicted; PRM^fSAD^ = parametric response mapping functional small airways disease; SD = standard deviation; SGRQ = St. George’s Respiratory Questionnaire score; TLC_CT_% = total lung capacity % predicted and 6-MWD = 6-min walk test.

**Supplementary Table S6.** Multivariable-adjusted associations between post-bronchodilator forced vital capacity /total lung capacity (FVC/TLC_CT_) quartiles at enrollment with chronic bronchitis, dyspnea and health-related quality of life scores, chest CT % emphysema and % gas trapping, functional small airway disease, and 6-min walk test distance in smokers with preserved ratio impaired spirometry defined based on the lower limit of normal (n=973).

|  | **FVC/TLC_CT_** | | | | |
| --- | --- | --- | --- | --- | --- |
| **Chronic Bronchitis** | OR | 2.5% | 97.5% | P value | |
| **Very High** | ref | ref | ref | ref | |
| **High** | 2.54 | 1.53 | 4.28 | <0.001 | |
| **Low** | 1.72 | 1.00 | 3.00 | 0.053 | |
| **Very Low** | 2.53 | 1.42 | 4.57 | 0.002 | |
| **mMRC** | coef | 2.5% | 97.5% | P value |  |
| **Very High** | ref | ref | ref | ref |  |
| **High** | 0.03 | -0.22 | 0.27 | 0.83 |  |
| **Low** | 0.03 | -0.23 | 0.29 | 0.82 |  |
| **Very Low** | 0.40 | 0.12 | 0.68 | 0.006 |  |
| **SGRQ** | coef | 2.5% | 97.5% | P value | |
| **Very High** | ref | ref | ref | ref | |
| **High** | 0.51 | -3.23 | 4.26 | 0.79 | |
| **Low** | 1.92 | -2.02 | 5.86 | 0.34 | |
| **Very Low** | 6.04 | 1.73 | 10.36 | 0.006 | |
| **% Emphysema** | coef | 2.5% | 97.5% | P value | |
| **Very High** | ref | ref | ref | ref | |
| **High** | 0.44 | -0.06 | 0.95 | 0.081 | |
| **Low** | 0.71 | 0.18 | 1.23 | 0.009 | |
| **Very Low** | 1.51 | 0.93 | 2.08 | <0.001 | |
| ***% Gas trapping** | coef | 2.5% | 97.5% | P value | |
| **Very High** | ref | ref | ref | ref | |
| **High** | 0.74 | -0.81 | 2.28 | 0.35 | |
| **Low** | 2.52 | 0.88 | 4.15 | 0.003 | |
| **Very Low** | 5.54 | 3.74 | 7.34 | <0.001 | |
| **^†^ %PRM^fSAD^** | coef | 2.5% | 97.5% | P value | |
| **Very High** | ref | ref | ref | ref | |
| **High** | 0.02 | -1.76 | 1.80 | 0.98 | |
| **Low** | 2.04 | 0.16 | 3.92 | 0.033 | |
| **Very Low** | 5.23 | 3.18 | 7.29 | <0.001 | |
| **^#^6-MWT distance, meters** | coef | 2.5% | 97.5% | P value | |
| **Very High** | ref | ref | ref | ref | |
| **High** | 11.57 | -48.54 | 71.67 | 0.71 | |
| **Low** | -20.45 | -83.53 | 42.62 | 0.52 | |
| **Very Low** | -36.44 | -105.74 | 32.85 | 0.30 | |

Binary logistic regression models with post-bronchodilator FVC/TLC_CT_ quartiles as independent variables (exposure) and chronic bronchitis as the dependent variables (outcome) were performed. Linear regression models with post-bronchodilator FVC/TLC_CT_ quartiles as independent variables (exposure) and mMRC, SGRQ, % Emphysema, % Gas trapping, PRM^fSAD^, and 6-MWT distance as the dependent variables (outcome) were performed. All models included the following co-variates: age, sex, race, body mass index, smoking status at the enrollment, smoking pack-years, history of asthma and congestive heart failure.

* For % GT analysis, data were available for 819 participants.

† For PRM^fSAD^data analysis, data were available for 806 participants.

#For 6-MWT distance analysis, data were available for 962 participants.

mMRC = modified Medical Research Council dyspnea score; OR=odds ratio; PRM^fSAD^= parametric response mapping functional small airways disease; SGRQ = St. George’s Respiratory Questionnaire score; 6-MWT = 6-min walk test.

**Supplementary Table S7.** Multivariable-adjusted associations between post-bronchodilator forced vital capacity /total lung capacity (FVC/TLC_CT_) quartiles at enrollment with change in FEV_1_, 6-MWT distance, % emphysema and gas trapping between enrollment and follow-up, and progression to COPD (defined as FEV_1_/FVC<LLN) at 5-year follow-up visit in smokers with preserved ratio impaired spirometry defined based on the lower limit of normal.

|  | **FVC/TLC_CT_** | | | |
| --- | --- | --- | --- | --- |
| **Change in FEV1, ml/year** | coef | 2.5% | 9.75% | P value |
| **Very High** | ref | ref | ref | ref |
| **High** | 1.70 | -11.45 | 14.84 | 0.80 |
| **Low** | 2.42 | -11.44 | 16.29 | 0.73 |
| **Very Low** | 5.57 | -9.64 | 20.78 | 0.47 |
| **Change in 6-MWT distance, meters/year** | coef | 2.5% | 9.75% | P value |
| **Very High** | ref | ref | ref | ref |
| **High** | -7.75 | -31.83 | 16.33 | 0.53 |
| **Low** | 9.28 | -16.08 | 34.65 | 0.47 |
| **Very Low** | 6.16 | -21.72 | 34.04 | 0.66 |
| **Change in % Emphysema per year** | coef | 2.5% | 9.75% | P value |
| **Very High** | ref | ref | ref | ref |
| **High** | -0.02 | -0.60 | 0.56 | 0.93 |
| **Low** | 0.01 | -0.63 | 0.64 | 0.98 |
| **Very Low** | 0.21 | -0.49 | 0.91 | 0.56 |
| **Change in % Gas trapping per year** | coef | 2.5% | 9.75% | P value |
| **Very High** | ref | ref | ref | ref |
| **High** | 1.19 | -0.79 | 3.18 | 0.24 |
| **Low** | 3.39 | 1.20 | 5.58 | 0.003 |
| **Very Low** | 2.53 | 0.03 | 5.03 | 0.047 |
| **COPD at 5 years** | OR | 2.5% | 9.75% | P value |
| **Very High** | ref | ref | ref | ref |
| **High** | 0.97 | 0.50 | 1.88 | 0.93 |
| **Low** | 1.63 | 0.85 | 3.15 | 0.15 |
| **Very Low** | 3.18 | 1.62 | 6.40 | <0.001 |

Linear regression models with post-bronchodilator FVC/ quartiles as independent variables (exposure) change in FEV1, 6-MWT distance, % emphysema and gas trapping between enrollment and follow-up as the dependent variables(outcomes) were performed. Binary logistic regression models with post-bronchodilator FVC/TLC_CT_ quartiles as independent variables (exposure) and progression to COPD at follow-up visit as the dependent variables (outcome) were performed. All models included the following co-variates: age, sex, race, body mass index, smoking status at the enrollment, smoking pack-years, history of asthma and congestive heart failure.

For change in FEV_1_, data were available for 530 participants.

For change in 6-MWT distance analysis, data were available for 521participants.

For change in % emphysema analysis, data were available for 420 participants.

For change in % gas trapping analysis, data were available for 325 participants.

OR= odds ratio; 6-MWT = 6-min walk test.

**Supplementary Table S8.** Multivariable-adjusted associations between post-bronchodilator forced vital capacity /total lung capacity (FVC/TLC_CT_) quartiles at enrollment with prospective total exacerbations and severe exacerbations in smokers with preserved ratio impaired spirometry defined based on the lower limit of normal (n= 851).

|  | **FVC/TLC_CT_** | | | |
| --- | --- | --- | --- | --- |
| **Exacerbations** | IRR | 2.5% | 9.75% | P value |
| **Very High** | ref | ref | ref | ref |
| **High** | 1.07 | 0.70 | 1.63 | 0.75 |
| **Low** | 1.07 | 0.69 | 1.65 | 0.77 |
| **Very low** | 2.23 | 1.44 | 3.45 | <0.001 |
| **Severe Exacerbations** | IRR | 2.5% | 9.75% | P value |
| **Very High** | ref | ref | ref | ref |
| **High** | 1.18 | 0.68 | 2.04 | 0.55 |
| **Low** | 1.32 | 0.74 | 2.36 | 0.34 |
| **Very Low** | 2.98 | 1.65 | 5.36 | <0.001 |

For exacerbation analysis, data for 851 of total 973 participants with PRISm were available. Zero-inflated negative binomial regression models with post-bronchodilator FVC/TLC_CT_ quartile as independent variables (exposure) and total exacerbations and severe exacerbations as the dependent variables (outcome) were performed. All regression models included the following co-variates: age, sex, race, body mass index, smoking status at the enrollment, smoking pack-years, history of asthma and congestive heart failure, and chronic bronchitis in the count negative binomial regression and an intercept-only model in the zero component. Follow-up time was included as an offset in the models.

IRR= incident rate ratio.

**Supplementary Table S9.** Multivariable-adjusted associations between post-bronchodilator forced vital capacity /total lung capacity (FVC/TLC_CT_) quartiles at enrollment with mortality in smokers with Preserved Ratio Impaired Spirometry defined based on the lower limit of normal (n=839).

|  | **FVC/TLC_CT_** | | | |
| --- | --- | --- | --- | --- |
| **Quartile** | **HR** | **2.5%** | **9.75%** | **P value** |
| **Very High** | ref | ref | ref | ref |
| **High** | 1.59 | 0.79 | 3.19 | 0.19 |
| **Low** | 1.78 | 0.89 | 3.57 | 0.10 |
| **Very Low** | 1.78 | 0.84 | 3.75 | 0.13 |

Cox Hazard regression models with post-bronchodilator FVC/TLC_CT_ quartiles as independent variables (exposure) and mortality as the dependent variable(outcome) were performed.

All models for mortality included the following co-variates: age, sex, race, smoking status, smoking pack-years, body mass index (BMI), history of asthma and congestive heart failure, and diabetes mellitus.

HR= Hazard Ratio

**Supplementary Figure S1.** Kaplan-Meier Plot of overall survival by forced vital capacity /total lung capacity ratio (FVC/TLC_CT_) quartiles at enrollment in smokers with Preserved Ratio Impaired Spirometry (PRISm) defined based on the lower limit of normal (n=839).


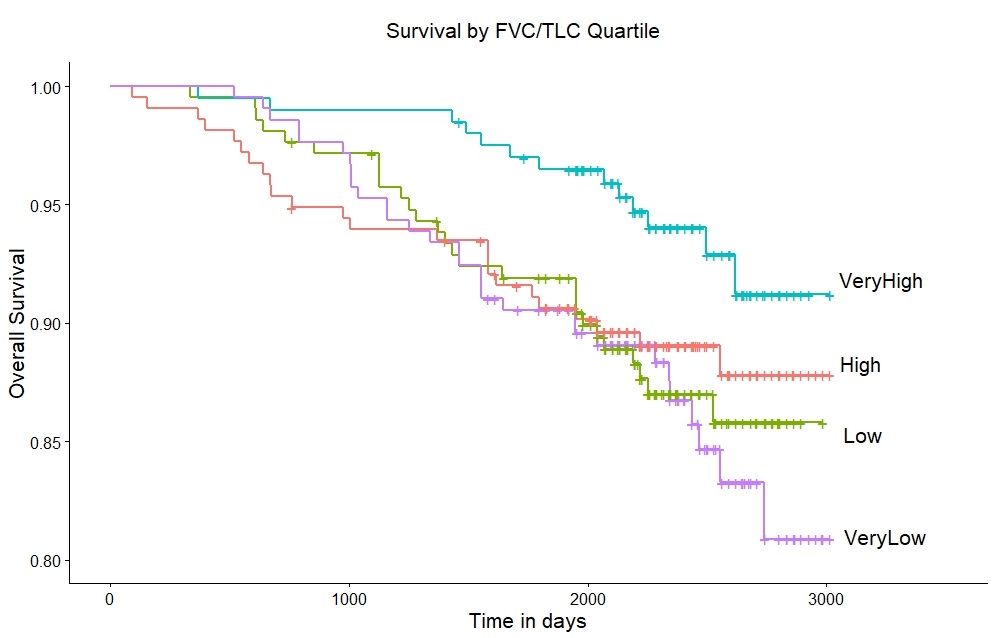


Chi-squared p-value for differences in mortality by quartile = 0.07.
